# Enhancing clinical decision-making in closed pelvic fractures with machine learning models

**DOI:** 10.17305/bb.2024.10802

**Published:** 2024-11-29

**Authors:** Dian Wang, Yongxin Li, Li Wang

**Affiliations:** 1 Department of Emergency, Sichuan Provincial People’s Hospital Chuandong Hospital, Dazhou First People’s Hospital, Tongchuan District, Dazhou, Sichuan Province, China; 2 Department of Critical Care Medicine, Suining Municipal Hospital of Traditional Chinese Medicine, Suining, Sichuan Province, China

**Keywords:** Hemodynamic instability, HI, closed pelvic fracture, PF, machine learning, ML, risk prediction, clinical decision-making, mortality risk

## Abstract

Closed pelvic fractures (PFs) can lead to severe complications, including hemodynamic instability (HI) and mortality. Accurate prediction of these risks is crucial for effective clinical management. This study aimed to utilize various machine learning (ML) algorithms to predict HI and death in patients with closed PFs and identify relevant risk factors. The retrospective study included 208 patients diagnosed with PFs and admitted to Suning Traditional Chinese Medicine Hospital between 2019 and 2023. Among these, 133 cases were identified as closed PFs. Patients with closed fractures were divided into a training set (*n* ═ 115) and a test set (*n* ═ 18). The training set was further stratified into two groups based on hemodynamic stability: Group A (patients with HI) and Group B (patients with hemodynamic stability). A total of 40 clinical variables were collected, and multiple ML algorithms were employed to develop predictive models, including logistic regression (LR), C5.0 decision tree, Naive Bayes (NB), support vector machine (SVM), K-nearest neighbors (KNN), random forest (RF), and artificial neural network (ANN). Additionally, factor analysis was performed to assess the interrelationships between variables. The RF and LR algorithms outperformed traditional methods—such as central venous pressure (CVP) and intra-abdominal pressure (IAP) measurements—in predicting HI. The RF model achieved an average area under the ROC curve (AUC) of 0.92, with an accuracy of 0.86, precision of 0.81, and an F1 score of 0.87. The LR model had an average AUC of 0.82 but shared the same accuracy, precision, and F1 score as the RF model. Key risk factors identified included TILE grade, heart rate (HR), creatinine (CR), white blood cell (WBC) count, fibrinogen (FIB), and lactic acid (LAC), with LAC levels >3.7 and an Injury Severity Score (ISS) >13 as significant predictors of HI and mortality. In conclusion, the RF and LR algorithms are effective in predicting HI and mortality risk in patients with closed PFs, enhancing clinical decision-making and improving patient outcomes.

## Introduction

Pelvic fractures (PFs), a common type of traumatic injury, pose a serious threat to patient safety [1]. Among these, closed PFs are particularly concerning due to the rich vascularity surrounding the pelvic bones, which increases the risk of severe internal bleeding and hemodynamic instability (HI) following a fracture [2–4]. Such cases often require urgent medical intervention to prevent fatal outcomes. However, the complexity and variability of closed PFs make it challenging for clinicians to accurately assess associated risks [5]. Current assessment methods primarily rely on intuitive clinical judgment and traditional monitoring of physiological parameters. While helpful for diagnosis, these methods have significant limitations in predicting HI and mortality risks. Traditionally, physicians evaluate the hemodynamic status of PF patients using central venous pressure (CVP) and intra-abdominal pressure (IAP) measurements [6–8]. Despite their widespread use, these methods are limited in their ability to predict long-term patient outcomes [9–11]. For example, CVP and IAP readings can be influenced by numerous factors, failing to reliably reflect the severity of HI [12]. Moreover, these approaches provide little direct insight into patients’ mortality risks [13–15]. As a result, there is an urgent need for advanced predictive tools that offer more accurate assessments, enabling clinicians to better understand patient conditions and make informed decisions. In recent years, machine learning (ML) technologies have gained significant attention in the medical field [16, 17]. By analyzing large volumes of clinical data, ML algorithms can identify key risk factors for diseases and predict their progression [18]. ML has shown considerable potential in prognostic analyses of closed PFs [19]. Algorithms, such as logistic regression (LR), decision trees (DTs), Naive Bayes (NB), support vector machines (SVMs), K-nearest neighbors (KNNs), random forest (RF), and artificial neural networks (ANNs), can process complex datasets and extract valuable insights [20–22]. These insights encompass patients’ physiological data and additional variables, such as age, gender, and injury severity, all of which can impact prognosis [23–25]. Through these tools, clinicians can achieve a more holistic understanding of patient conditions and make more accurate predictions. This study aims to leverage advanced ML algorithms to predict HI and mortality risks in patients with closed PFs. We collected extensive clinical data and applied seven ML algorithms to identify the factors most relevant to these risks. Compared to traditional evaluation methods, this approach provides more precise and comprehensive risk predictions. Additionally, the findings of this study will help clinicians more accurately identify high-risk patients, enabling timely and targeted interventions. This not only improves patient survival rates but also enhances their long-term prognoses. In conclusion, our research highlights the significant potential of ML in advancing the accuracy of prognosis analysis for closed PFs. This holds critical scientific and clinical importance in refining treatment strategies and improving patient outcomes.

## Materials and methods

### Study design and grouping

**Figure 1.**
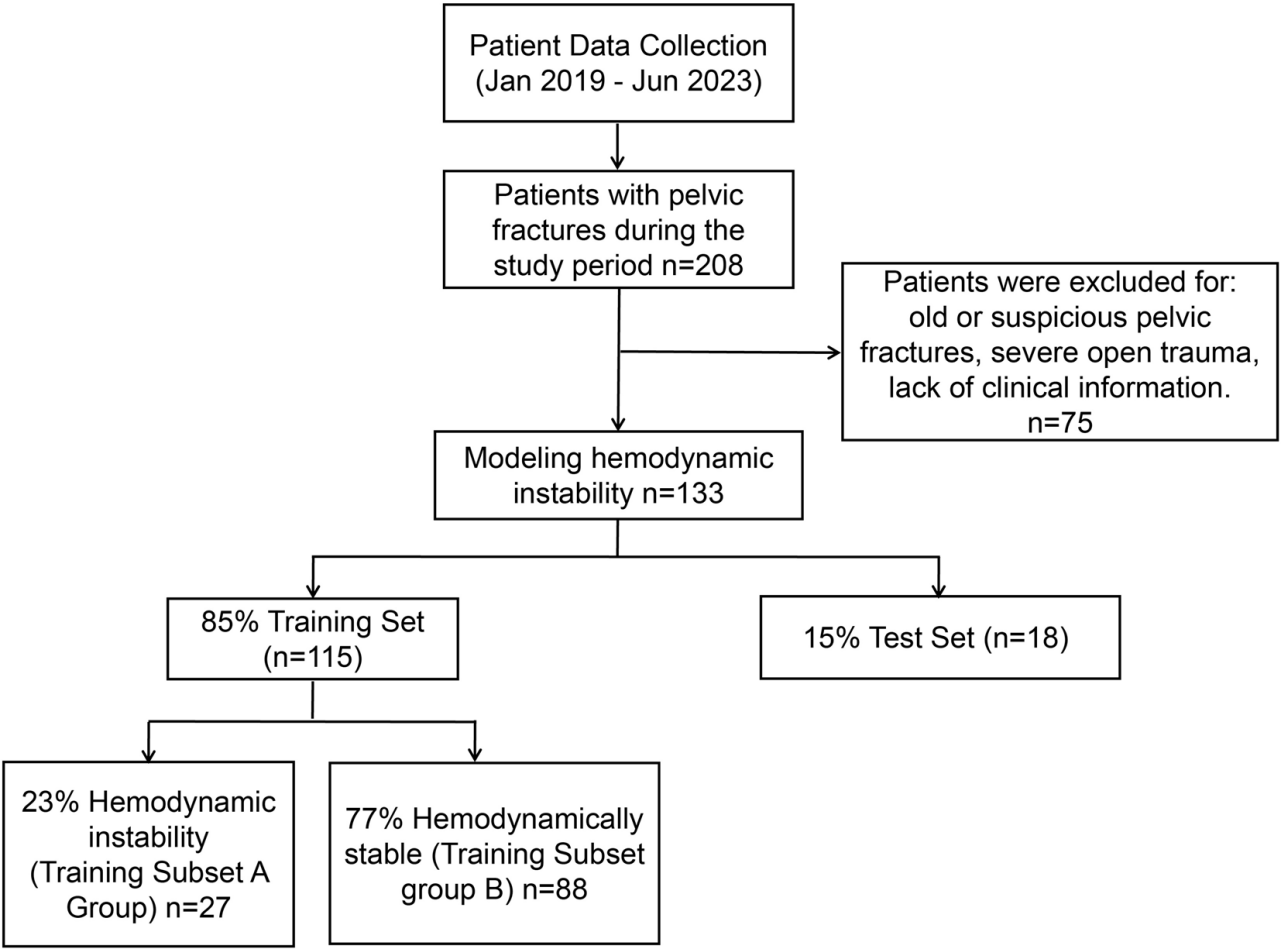
**Research design and grouping process.** CVP: Central venous pressure; IAP: Intra-abdominal pressure.

From January 2019 to June 2023, this study recruited 208 patients hospitalized for PFs at Suining Traditional Chinese Medicine Hospital in Sichuan Province. The inclusion criteria were based on the Fractures (Complex): Assessment and Management (2016) guidelines from the National Clinical Guideline Center. To qualify, patients needed a clear history of trauma, local swelling, bruising, pain, positive pelvic compression and separation test results, and radiographic or CT evidence of PFs and displacement. All 208 patients initially met these criteria and were confirmed to have PFs. After thoroughly reviewing each patient’s medical records and imaging results, 75 patients who did not meet the criteria for closed PFs were excluded. Excluded cases included patients with old fractures (those showing signs of healing), suspected PFs unconfirmed by imaging, and complex cases involving non-blunt trauma, such as penetrating injuries or burns. Ultimately, 133 patients with closed PFs were included in the study. The included patients were randomly assigned to either a training set (115 cases) or a test set (18 cases) using the numpy.random.permutation function from Python’s numpy.random module, which generated an unbiased random sequence to shuffle the patient list. This randomization allocated 85% of the patients to the training set and 15% to the test set. To protect privacy, we ensured data completeness and de-identified all patient information. Within the training set, patients were further divided into two subsets based on hemodynamic status: Training Subset A (27 patients with HI) and Training Subset B (88 patients with hemodynamic stability). Patients in Training Subset A exhibited severe clinical conditions, including a systolic blood pressure (SBP) below 90 mmHg upon admission, a need for blood transfusions or vasopressor support to maintain blood pressure, a base deficit greater than 6 mmol/L, a shock index above 1, and the requirement of at least 4–6 units of packed red blood cells. In contrast, patients in Training Subset B (hemodynamically stable) presented more stable clinical conditions, such as normal SBP, no need for transfusions or vasopressor support, a base deficit not exceeding 6 mmol/L, a shock index below 1, and good cardiac output with stable hemodynamics. To validate the model’s accuracy, the remaining 15% of patients (18 cases) from the randomly assigned test set were used. The detailed inclusion and exclusion process is illustrated in Figure 1. Given the limited sample size, we employed cross-validation techniques (e.g., five-fold cross-validation) and L2 regularization to reduce the risk of model overfitting. Cross-validation maximizes data utilization by training and validating the model on multiple subsets, enhancing its generalization capability.

This retrospective analysis explores the clinical characteristics and treatment outcomes of patients with closed PFs, strictly adhering to medical ethical principles, laws, and regulations. Patient data were sourced from the medical records of the Traditional Chinese Medicine Hospital in Suining City, Sichuan Province, with all information de-identified beforehand to protect privacy and ensure information security. Since the study is retrospective and does not involve direct patient intervention, patient-informed consent was not required. Additionally, the study received approval from the hospital’s ethics review board, ensuring compliance with ethical standards. The research team remains committed to conducting all data collection and analysis with scientific integrity and robust data protection measures, guaranteeing the authenticity and reliability of the findings.

### Collection and data cleaning of clinical variables for 133 patients with closed PFs

**Figure 2.**
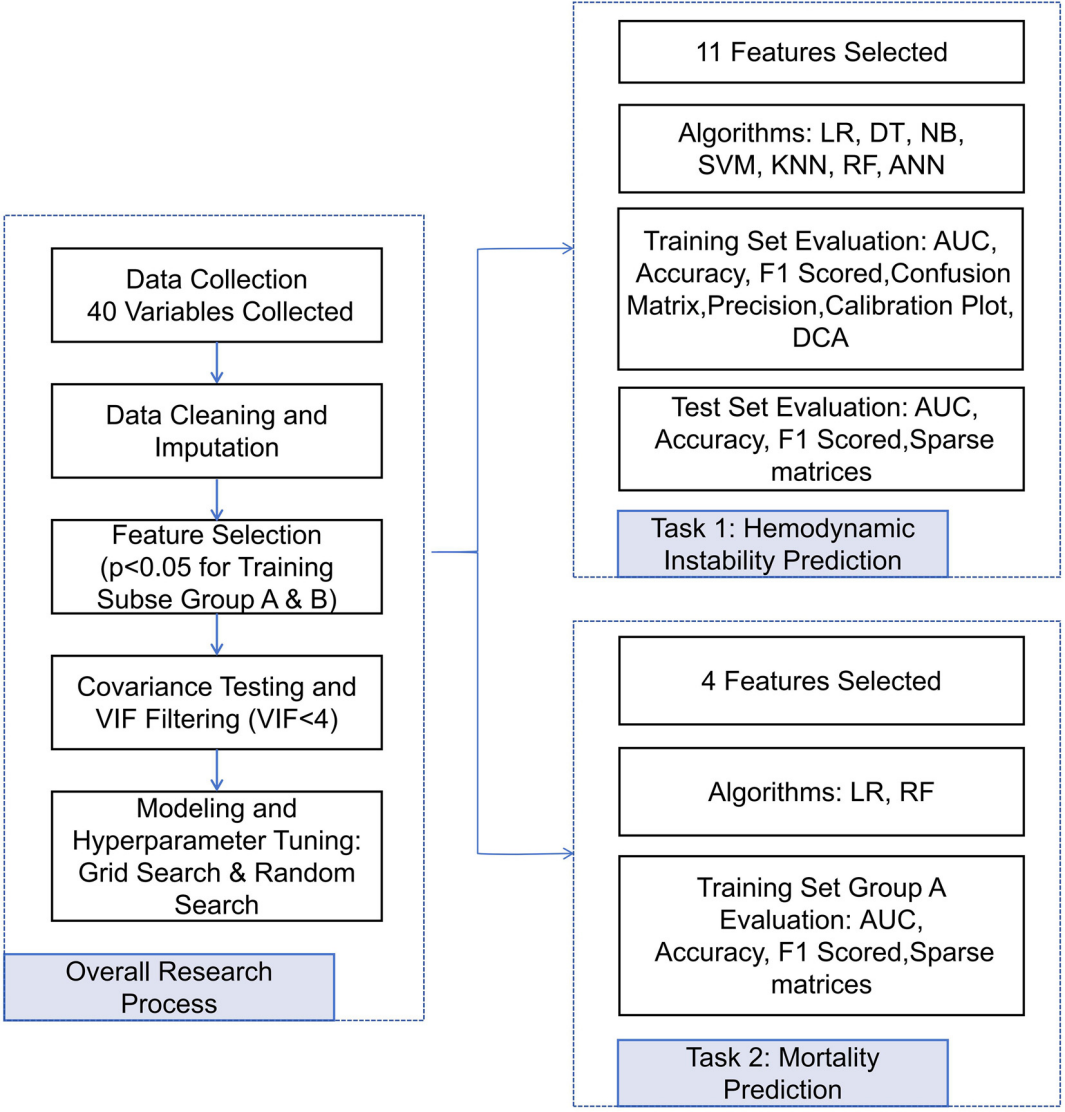
**Flowchart of the research methods**. LR: Logistic regression; C5.0: C5.0 decision tree algorithm; NB: Naive Bayes; SVM: Support vector machine; KNN: K-nearest neighbors; RF: Random forest; ANN: Artificial neural network; VIF: Variance in ation factor; AUC: Area under the ROC curve; DT: Decision tree.

In this study, we collected 40 clinical variables by building on previous research and integrating clinical practice [26–29]. These variables included general patient information (e.g., gender and age), the cause of injury, and vital signs upon admission (body temperature [T], heart rate [HR], blood pressure, and respiratory rate). Additional assessments included arterial blood gas analysis upon admission (hydrogen ion concentration [pH], partial pressure of carbon dioxide [pCO_2_], partial pressure of oxygen [pO_2_], base excess [BE], and lactic acid [LAC]), routine blood tests (white blood cell count [WBC], red blood cell count [RBC], platelets [PLT], and hemoglobin [HGB]), coagulation function (prothrombin time [PT], prothrombin time activity percentage [PT%], international normalized ratio [INR], fibrinogen [FIB], and thrombin time [TT]), as well as liver and kidney function indicators (alanine transaminase [ALT], aspartate aminotransferase [AST], albumin [ALB], and creatinine [CR]). Disease severity was assessed using various scoring systems, including the Glasgow Coma Scale (GCS), Injury Severity Score (ISS), Revised Trauma Score (RTS), Trauma and ISS(TRISS), Acute Physiology and Chronic Health Evaluation II (APACHE II), World Society of Emergency Surgery (WSES) classification, and the Tile scoring system. Treatment indicators encompassed the use of vasopressors and transfusion status within the first 24 h. To handle missing or incomplete data, we performed data imputation and ensured the removal of values deemed implausible in clinical practice. Missing values for continuous variables were imputed using the mean, while missing values for categorical variables were assigned to a distinct “missing data” category [30, 31]. The dataset was also standardized for uniformity. We conducted covariance tests and univariate LR analyses on these clinical variables. Feature selection required meeting two criteria: (1) a statistically significant difference between training subsets A and B (*P* < 0.05) and (2) no significant difference between the training and test sets (*P* > 0.05). As a result, 11 feature variables were identified with HI as the outcome, while four feature variables were selected for model training and validation with mortality as the outcome. A detailed methodological flowchart is presented in Figure 2.

### Data processing and sampling strategy

The Synthetic Minority Over-Sampling Technique (SMOTE) is a method used to enhance the representation of minority classes by synthesizing new samples. In this study, SMOTE was applied to the relatively rare samples of hemodynamically unstable patients in the training set. The process involved selecting a sample from the minority class and its nearest neighbors, then performing linear interpolation between them to generate new data points. This approach increased the size of the training set and improved sample diversity, enabling the model to learn minority class features more comprehensively.

To further balance the class distribution, we also employed a random undersampling strategy, which reduces the majority class by randomly removing a portion of its samples. Since the training set contained a higher abundance of hemodynamically stable patient samples, random undersampling was applied to this class. While this method is straightforward, it effectively minimizes bias toward the majority class. However, it requires careful execution to avoid the loss of critical information. Notably, these sampling techniques were applied only during the training phase. To optimize the model and enhance its generalizability, we utilized five-fold cross-validation. For the test set, we maintained the original sample distribution without applying any sampling adjustments, ensuring the independence and reliability of the test results.

### Data splitting and cross-validation

We randomly divided the patients into a training set (85%) and a test set (15%) using Python’s numpy.random.permutation function to ensure unbiased and random sample allocation. To prevent overfitting and enhance the robustness of the predictive model, we employed a five-fold cross-validation approach. In this method, the training set is divided into five subsets. Each subset takes turns serving as the validation set, while the remaining four subsets are used as training data. This process is repeated five times, ensuring that each subset serves as the validation set exactly once. We evaluate the model’s performance and stability by averaging the results across these five iterations, allowing every data point to participate in both training and validation. This approach helps assess the model’s consistency across different data subsets, ultimately improving its generalizability and reliability.

### Systematic hyperparameter tuning

To ensure optimal model performance, we use two primary methods for adjusting model parameters: Grid Search and Random Search. Grid Search systematically evaluates all possible combinations of specified parameters to identify the best configuration, while Random Search selects parameter combinations at random within the defined parameter space. Although Random Search is less exhaustive than Grid Search, it is often more efficient for exploring large parameter spaces. For LR, the key parameters to optimize are the regularization strength (C: [0.01, 0.1, 1, 10, 100]) and the type of regularization penalty ([l1, l2]). In the case of the C5.0 DT model (C5.0), optimization focuses on the tree depth (max_depth: [3, 5, 10, 20]) and the minimum number of samples required at a leaf node (min_samples_leaf: [1, 2, 5, 10]). For NB, tuning involves adjusting the smoothing parameter (var_smoothing: [1e-9, 1e-8, 1e-7]) across different variations, such as Gaussian NB. For SVM, the optimization parameters include the kernel type ([linear, poly, RBF, sigmoid]), the regularization parameter (C: same as LR), and the kernel function parameter (gamma: [scale, auto, 0.1, 1, 10]). With KNN, fine-tuning involves the number of neighbors (n_neighbors: [3, 5, 7, 10]) and the distance metric ([euclidean, manhattan]). In the RF model, the parameters to optimize are the number of trees (n_estimators: [100, 200, 300, 400, 500]), the maximum depth of the trees (max_depth: [10, 20, 30, None]), and the minimum number of samples required to split a node (min_samples_split: [2, 5, 10, 15]). Lastly, for ANN, optimization focuses on the number of layers ([1, 2, 3, 5]), the number of neurons per layer ([10, 50, 100, 200]), and the learning rate ([0.001, 0.01, 0.1]).

### Construction and validation of an HI prediction model: A comprehensive analysis of multiple algorithms

In this study, we utilized 11 feature variables, encompassing both categorical and continuous types, to develop a model for predicting a binary outcome. We employed seven supervised learning methods [32, 33]. LR: Models the relationship between input features and output types to predict new sample classifications; C5.0 DT: Constructs a tree by sequentially splitting input features based on partitions that maximize information gain; SVM: Identifies the optimal hyperplane that separates data types as the decision boundary; KNN: Predicts unknown sample classifications based on the K nearest known samples, using distance metrics like Euclidean or Manhattan; ANN: Uses interconnected neurons to process input signals through activation functions and generate predictions; NB: Employs probabilistic methods based on Gaussian distribution assumptions; and RF: Builds multiple DTs by randomly selecting features, where each tree predicts an outcome, and the final prediction is determined via majority voting. To address data imbalance and improve model generalizability, we applied SMOTE and random undersampling strategies within the training set. Additionally, we utilized five-fold cross-validation to optimize model parameters. These sampling techniques were restricted to the training phase, while the test set retained its original distribution to ensure an independent and accurate evaluation. To further analyze model performance, we conducted a factor analysis on the model outputs to identify the variables that contributed most to the predictions. The test set was also used for supplementary analysis, as depicted in Figure 2.

### Construction and validation of a risk prediction model for mortality in HI patients: An analysis based on LR and RF

We analyzed data from 27 hemodynamically unstable patients in training subset A, using four feature variables to model and predict mortality. Supervised learning methods, specifically LR and RF algorithms, were employed to build the predictive model, demonstrating strong overall performance during validation with internal data. Key model variables were identified through factor analysis, and their optimal cutoff values were determined to enhance predictive accuracy. The LR algorithm established a probabilistic relationship between input features and outcomes (i.e., survival or death), allowing it to predict mortality risk. In contrast, the RF algorithm improved prediction accuracy and stability by constructing multiple DTs and consolidating their outputs. During model training, we applied cross-validation on training subset A to ensure reliability and robustness. Factor analysis was used to isolate critical variables influencing prediction outcomes, with cutoff values optimized to further enhance performance. This comprehensive approach enabled the development of a validated model capable of accurately predicting mortality risk in this patient population. To assess model performance on clinical samples, we used key evaluation metrics [34–36], including the confusion matrix, the receiver operating characteristic (ROC) curve, and the F1 score. The confusion matrix captured classification results (true positives, false positives, true negatives, and false negatives), while the ROC curve illustrated the trade-off between true positive and false positive rates across thresholds. The F1 score balanced precision and recall, providing a holistic view of performance. Additionally, accuracy, defined as the proportion of correct predictions, was calculated as a straightforward measure of the model’s overall effectiveness. Precision (true positives as a proportion of predicted positives) was also emphasized to evaluate the quality of positive predictions. To explore clinical utility, we employed calibration plots and decision curve analysis (DCA). Calibration plots showed the agreement between predicted probabilities and actual outcomes, with ideal performance aligning closely to the diagonal. DCA measured the net benefit of the model across various thresholds, helping compare the utility of the model’s predictions to baseline strategies, such as treating all patients or taking no action. We also validated diagnostic accuracy by comparing CVP and Intra-IAP at patient admission. Using the Youden Index—defined as sensitivity minus (1-specificity)—we calculated optimal cutoff values to refine the model’s predictive performance. Factor analysis again highlighted the critical variables driving mortality risk prediction, underscoring the strength of our methodology. In conclusion, this study successfully developed and validated a predictive model for patient mortality risk through rigorous data analysis and advanced ML techniques.

### Ethical statement

This study was approved by the Clinical Ethics Committee of Suining Municipal Hospital of Traditional Chinese Medicine.

### Statistical analysis

This study utilized Python 3.7 (64-bit) and Anaconda Jupyter to execute scripts and conduct all statistical analyses and modeling. The NumPy and Pandas libraries were used for numerical computations and array operations. Continuous variables were expressed as either mean ± standard deviation or median with interquartile range, while categorical variables were summarized as frequencies. Normality testing of the data was conducted using the warnings library. Statistical tests, including Student’s *t*-test, Mann–Whitney *U* test, and chi-squared test, were performed using the SciPy library. LR analysis was carried out using the StatsModels library. In addition to assessing statistical significance (*P* value < 0.05) and effect size, information gain and error minimization criteria were considered. Univariate LR analyses were performed for each independent variable to evaluate their contribution to the target variable regarding information gain and impact on model error. Information gain and error minimization were selected as evaluation metrics, with a detailed recording of each variable’s contribution. During the model construction process, we performed variable selection to address multicollinearity issues and identified a set of significant key variables (e.g., HR, SBP, and WSES scores). The selection of these variables helps control model complexity and reduces the likelihood of overfitting. Based on the analysis, variables contributing the most to the model were selected, prioritizing those with high information gain and significant error reduction. This approach aimed to identify factors associated with the risk of HI. To further optimize variable selection, stepwise regression and regularization techniques, such as Lasso regression, were employed. Regularization methods effectively reduced unnecessary model complexity by introducing penalty terms, addressing multicollinearity, and enhancing the stability and generalization capability of the predictive model. Model construction utilized several algorithms, including LR, DT classifier, RF classifier, Gaussian NB, SVM, KNN, and multilayer perceptron classifier. To evaluate the model’s performance, the ROC curve was plotted using the Matplotlib library. Predictive accuracy was assessed via the area under the ROC curve (AUC), computed using the roc_auc_score function from the Scikit-learn library. Model calibration was assessed through linear regression analysis, treating predicted values as the independent variable and actual outcomes as the dependent variable. The slope and intercept of the linear fit were calculated to evaluate trends (slope) and baseline bias (intercept) in predictions. The Bonferroni correction method was applied to adjust *P* values, controlling the overall Type I error rate. The false discovery rate (FDR) control method was also implemented, especially during CM feature selection in ML models. FDR control maintained the discovery rate while controlling the proportion of false discoveries. All statistical tests were two-tailed, and a *P* value less than 0.05 was considered statistically significant.

## Results

### Analysis of baseline characteristics in patients

In this study, 133 patients were analyzed. Age and gender differences between training subsets A and B were assessed. The average age was 54.50 years in subset A and 61.67 years in subset B, with no statistically significant difference (*P* ═ 0.085). Similarly, gender distribution showed 48.1% males and 51.9% females in subset A, compared to 60.2% males and 39.8% females in subset B, which was also not statistically significant (*P* ═ 0.277). These results suggest the sample demonstrates good representativeness and balance in baseline characteristics, such as age and gender (Table 1).

### Clinical variable selection and analysis

This study screened 40 clinical variables, focusing particularly on significant differences between the high-dynamic stability group and the instability group, including HR, blood pressure (SBP and diastolic blood pressure [DBP]), WBC count, and APACHE II score. For instance, notable differences were observed in the APACHE II score (median of 12.5 in training subset A vs 8.0 in training subset B) and WBC count (mean of 14.3 in training subset A vs 8.3 in training subset B), (both with *P* values of 0.000). SBP and DBP also showed significant differences between the two groups (both with *P* values of 0.000). Additionally, these variables demonstrated significant differences between the non-survival and survival groups, suggesting their potential association with HI and mortality risk (Table 1).

### Selection of key clinical variables for predicting HI and mortality risk in HI patients

Covariance testing and univariate LR analysis were conducted on the clinical variables. Selection criteria required a statistically significant difference between training subsets A and B (*P* < 0.05) but no significant difference between the training and test sets (*P* > 0.05). For predicting HI, 11 feature variables were identified: HR, SBP, DBP, WBC, Tile classification, FIB, CR, pH, pCO_2_, pO_2_, and lactate (LAC). These variables demonstrated a variance inflation factor (VIF) > 4, with no significant difference (*P* > 0.05), indicating suitability for predicting HI (Table 2). For mortality prediction, four variables were selected: ISS, GCS, TRISS, and APACHE II. Each of these also had a VIF > 4 with no significant difference (*P* > 0.05), indicating relevance for predicting mortality risk (Table 2).

**Table 1:** Comparative clinical and laboratory data analysis between Groups A (HI) and B (HS)

**Variable**	**Training subset Group A (*n* ═ 27) (HI)**	**Training subset B Group (*n* ═ 88) (HS)**	***P* value**	**Death *P* value**	**Variable**	**Training subset Group A (*n* ═ 27) (HI)**	**Training subset Group B (*n* ═ 88) (HS)**	***P* value**	**Death *P* value**
Age (years)	54.50 ± 24.33	61.67 ± 16.54	0.085	0.389	RTS	7.84 (5.97–7.84)	7.84 (5.97–7.84)	0.046	0.000
Sex			0.277	0.071	TRISS	3.42 (0.52–4.88)	3.74 (−2.40–.88)	0.011	0.000
Male	13 (48.10)	53 (60.20)			APACHE II	12.5 (3.00–1700)	8.00 (0.00–1.00)	0.000	0.000
Female	14 (51.90)	35 (39.80)			Tile			0.018	0.126
Hypertension			0.556	1.000	A	2 (7.40)	25 (28.40)		
Yes	3 (11.10)	16 (18.20)			B	12 (44.40)	42 (47.70)		
No	24 (88.90)	72 (81.80)			C	13 (48.10)	21 (23.90)		
T (^∘^C)	36.50 (36.00–36.90)	36.50 (36.00–37.70)	0.589	0.779	WBC (10^9^/L)	14.3	8.3	0.001	0.058
HR (bpm)	84.50 (58–157)	80.00 (53–113)	0.007	0.305	RBC (10^9^/L)	3.76	3.83	0.719	0.414
Breath (bpm)	20 (11–33)	20 (16–25)	0.813	0.378	HGB (g/L)	113.81 ± 22.68	116.02 ± 19.09	0.590	0.899
SBP (mmHg)	112.16 ± 23.65	133.76 ± 21.84	0.000	0.106	PLT (10^9^/L)	156 (0.85–1.47)	138 (0.85–1.47)	0.494	0.752
DBP (mmHg)	69.81 ± 14.18	79.12 ± 10.38	0.000	0.206	PT (s)	12.55 (10.40–21.90)	11.80 (10.20–16.90)	0.026	0.265
Fracture site			0.687	0.026	PT%	89.40 (34.20–110.70)	93.40 (49.90–128.50)	0.019	0.530
Pubis	3 (11.10)	18 (20.5)			INR	1.09 (0.87–1.95)	1.01 (0.85–1.47)	0.019	0.177
Ischium	0 (0.00)	1 (1.10)			APTT (s)	25.55 (5.59–17.70)	26.70 (18.40–67.70)	0.137	0.188
Iliac crest	1 (3.70)	5 (5.70)			FIB (g/L)	1.98 (1.20–3.19)	2.52 (1.01–7.02)	0.000	0.024
Sacrum	0 (0.00)	1 (1.10)			TT (s)	17.25 (14.70–80.30)	16.30 (13.20–30.90)	0.008	0.528
Multisite	23 (85.2)	63 (71.60)			ALT (U/L)	44.50 (11.00–570.00)	25.00 (6.00–114.00)	0.000	0.000
WSES			0.000	0.000	AST (U/L)	57.50 (20.00–750.00)	34.00 (14.00–145.00)	0.000	0.001
Mild	0 (0.00)	22 (25.00)			ALB (g/L)	33.80 (20.50–41.20)	36.30 (12.00–49.60)	0.033	0.072
Moderate	1 (3.70)	66 (75.00)			UREA (mmol/L)	5.44 (2.40–16.37)	5.95 (2.07–39.60)	0.788	0.246
Severe	26 (96.3)	0 (0.00)			CR (umol/L)	68.00 (38.70–133.40)	58.50 (30.60–194.00)	0.03	0.937
Young Burgess			0.211	0.274	pH	7.22 (7.08–7.47)	7.48 (7.21–7.64)	0.000	0.008
LC	1 (3.70)	4 (4.50)			pCO_2_ (mmHg)	36.50 (21.00–95.00)	32.00 (16.00–61.00)	0.047	0.136
APC	1 (3.70)	18 (20.50)			pO_2_ (mmHg)	97.32 ± 38.13	115.74 ± 36.86	0.017	0.064
VS	3 (11.10)	10 (11.40)			LAC (mmol/L)	2.80 (0.60–16.40)	1.60 (0.70–14.80)	0.006	0.993
CM	22 (81.50)	56 (63.6)			BE (mmol/L)	−6.27 (5.80–20.20)	1.3 (−5.40–22.10)	0.000	0.000
Etiology			0.092	0.655	24 h blood transfusion volume	2 (0–2)	4 (0–6)	0.000	0.000
Car accident	17 (61.30)	37 (42.00)			Pressor agent			0.313	0.003
High fall injury	2 (7.40)	26 (29.50)			Yes	9 (33.30)	20 (22.70)		
Falling flat	6 (22.20)	21 (23.90)			No	18 (66.70)	68 (77.30)		
Bruise	2 (7.40)	4 (4.50)							
ISS	17.50 (4.00–57.00)	10.00 (4.00–34.00)	0.000	0.000					
GCS	13.00 (6.00–15.00)	14.00 (5.00–15.00)	0.000	0.000					

HI: Hemodynamic instability; WBC: White blood cell count; HR: Heart rate; SBP: Systolic blood pressure; DBP: Diastolic blood pressure; FIB: Fibrinogen; CR: Creatinine; pH: Hydrogen ion concentration; pCO_2_: Partial pressure of carbon dioxide; pO_2_: Partial pressure of oxygen; LAC: Lactic acid; GCS: Glasgow Coma Scale; ISS: Injury Severity Score; TRISS: Trauma and Injury Severity Score; APACHE II: Acute Physiology and Chronic Health Evaluation; WSES: World Society of Emergency Surgery; BE: Base excess; ALT: Alanine transaminase; AST: Aspartate aminotransferase; ALB: Albumin; TT: Thrombin time; INR: International normalized ratio; PT: Prothrombin time; PT%: Prothrombin time activity percentage; RBC: Red blood cell count; HGB: Hemoglobin; RTS: Revised Trauma Score; PLT: Platelets.

**Table 2:** Univariate logistic regression analysis of HI and mortality risk factors

**Variable**	**HI VIF**	**HI *P* value**	**Death VIF**	**Death *P* value**
Heart rate (HR)	**2.495**	**0.031**	**1.975**	**0.111**
Systolic blood pressure (SBP)	**2.281**	**0.000**	**3.723**	**0.181**
Diastolic blood pressure (DBP)	**2.179**	**0.000**	**2.841**	**0.189**
World Society of Emergency Surgery	19.581	0.000	7.631	0.000
White blood cells (WBCs)	**2.312**	**0.002**	**60.019**	**0.002**
Tile classification	**3.313**	**0.021**	**1.096**	**0.010**
Prothrombin time activity percentage	13.009	0.057	10.211	0.130
Fibrinogen (FIB)	**2.240**	**0.001**	**1.478**	**0.750**
Thrombin time	1.947	0.214	1.570	0.214
Alanine transaminase	20.925	0.002	10.631	0.005
Albumin	1.936	0.028	10.123	0.045
Creatinine (CR)	**3.723**	**0.025**	**14.469**	**0.025**
Pondus hydrogenii (pH)	**1.967**	**0.000**	**169.523**	**0.000**
Partial pressure of carbon dioxide (pCO_2_)	**1.889**	**0.001**	**75.287**	**0.113**
Partial pressure of oxygen (pO_2_)	**104.124**	**0.062**	**80.832**	**0.091**
Pappenheimer O_2_	1.768	0.022	10.760	0.022
Lactic acid (LAC)	**2.012**	**0.001**	**6.015**	**0.488**
Base excess	5.241	0.000	36.886	0.000
24 h blood transfusion volume	4.669	0.000	61.559	0.832
Pressor agent	2.179	0.313	12.563	0.200
Injury Severity Score (ISS)	**4.445**	**0.000**	**2.862**	**0.000**
Glasgow Coma Scale (GCS)	**7.137**	**0.001**	**1.037**	**0.000**
Revised Trauma Score	2.394	0.171	39.537	0.999
Trauma and Injury Severity Score (TRISS)	**4.265**	**0.039**	**3.091.**	**0.002**
Acute Physiology and Chronic Health Evaluation (APACHE II)	**4.028**	**0.000**	**2.179**	**0.000**

HI VIF refers to the VIF for HI, indicating the level of multicollinearity among the variables predicting HI. HI *P* value: The *P* value in statistical hypothesis testing related to high visibility inflation factor. Death VIF refers to the VIF for Death, assessing multicollinearity in the variables predicting mortality risk. Death *P* value: The *P* value in statistical hypothesis testing related to mortality variables. Baseline Information: In the cohort of 133 closed pelvic fracture patients, a total of ten deaths were reported. Univariate regression analysis was conducted to assess various risk factors related to HI and mortality, as shown in Table 1. HI: Hemodynamic instability; VIF: Variance inflation factor.

**Figure 3.**
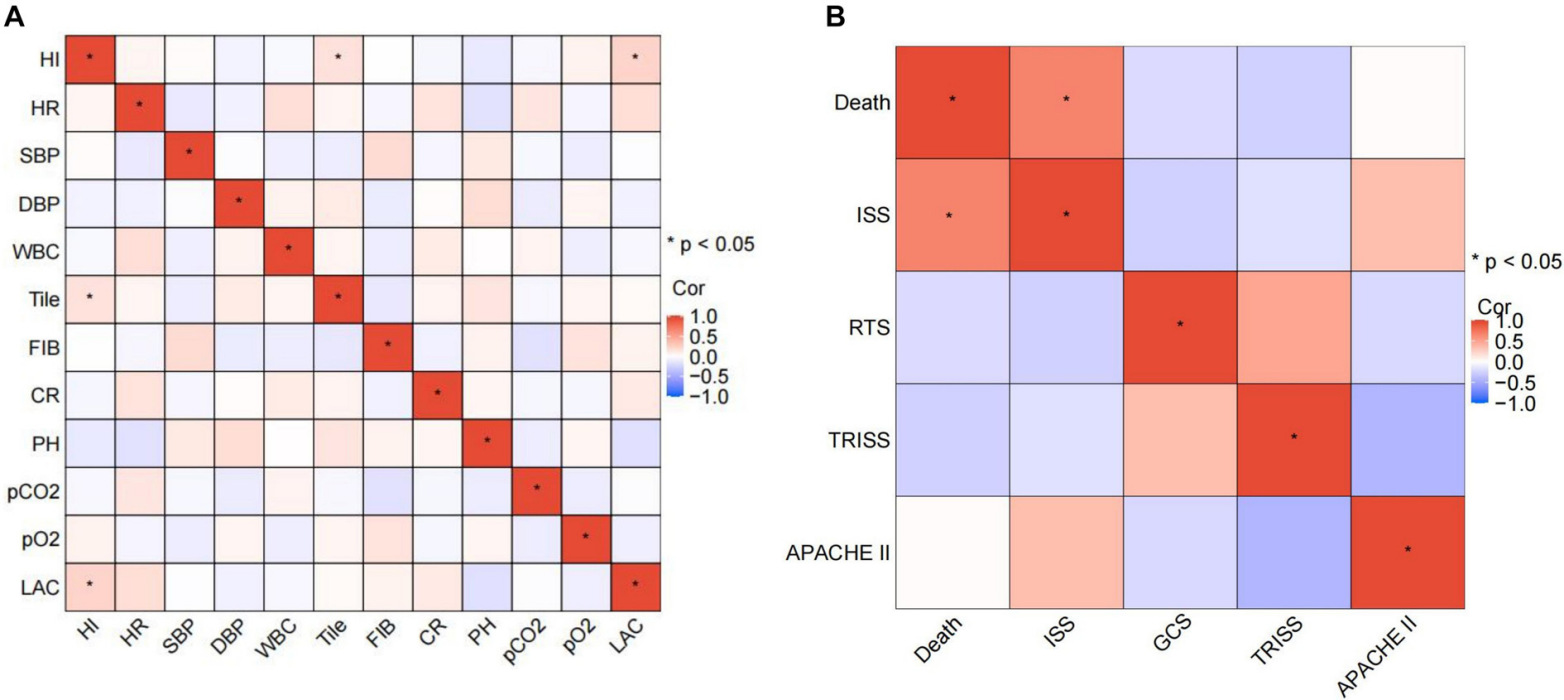
**Performance evaluation of the HI prediction model on the test set.** (A) Spearman correlation analysis was conducted on clinical data from 115 patients in the training set, including HI, HR, SBP, DBP, WBC, Tile classification, FIB, CR, pH, pCO_2_, pO_2_, and lactate (LAC) to assess correlations among clinical characteristics; (B) Spearman correlation analysis was conducted on the ISS, GCS, TRISS, and APACHE II in Group A of the training subset to analyze correlations among these variables for predicting mortality in HI patients. HI: Hemodynamic instability; WBC: White blood cell count; HR: Heart rate; SBP: Systolic blood pressure; DBP: Diastolic blood pressure; FIB: Fibrinogen; CR: Creatinine; pCO_2_: Partial pressure of carbon dioxide; pO_2_: Partial pressure of oxygen; LAC: Lactic acid; GCS: Glasgow Coma Scale; ISS: Injury Severity Score; TRISS: Trauma and Injury Severity Score; APACHE II: Acute Physiology and Chronic Health Evaluation; RTS: Revised Trauma Score.

We further evaluated the correlations among these variables using Spearman’s correlation analysis. Results showed no significant correlations among variables in the HI model, supporting their independence and suitability for further analysis and ensuring robust model predictiveness (Figure 3A). Tile classification and LAC were significantly positively correlated with HI (*P* < 0.05). In the mortality prediction model, no significant correlations were observed among the variables; however, ISS showed a positive correlation with mortality in HI patients (*P* < 0.05), while GCS and TRISS were negatively correlated with mortality, and APACHE II showed no significant association (*P* > 0.05), indicating no statistical significance (Figure 3B).

### Application of ML in predicting HI: Model comparison and key indicator analysis

**Figure 4.**
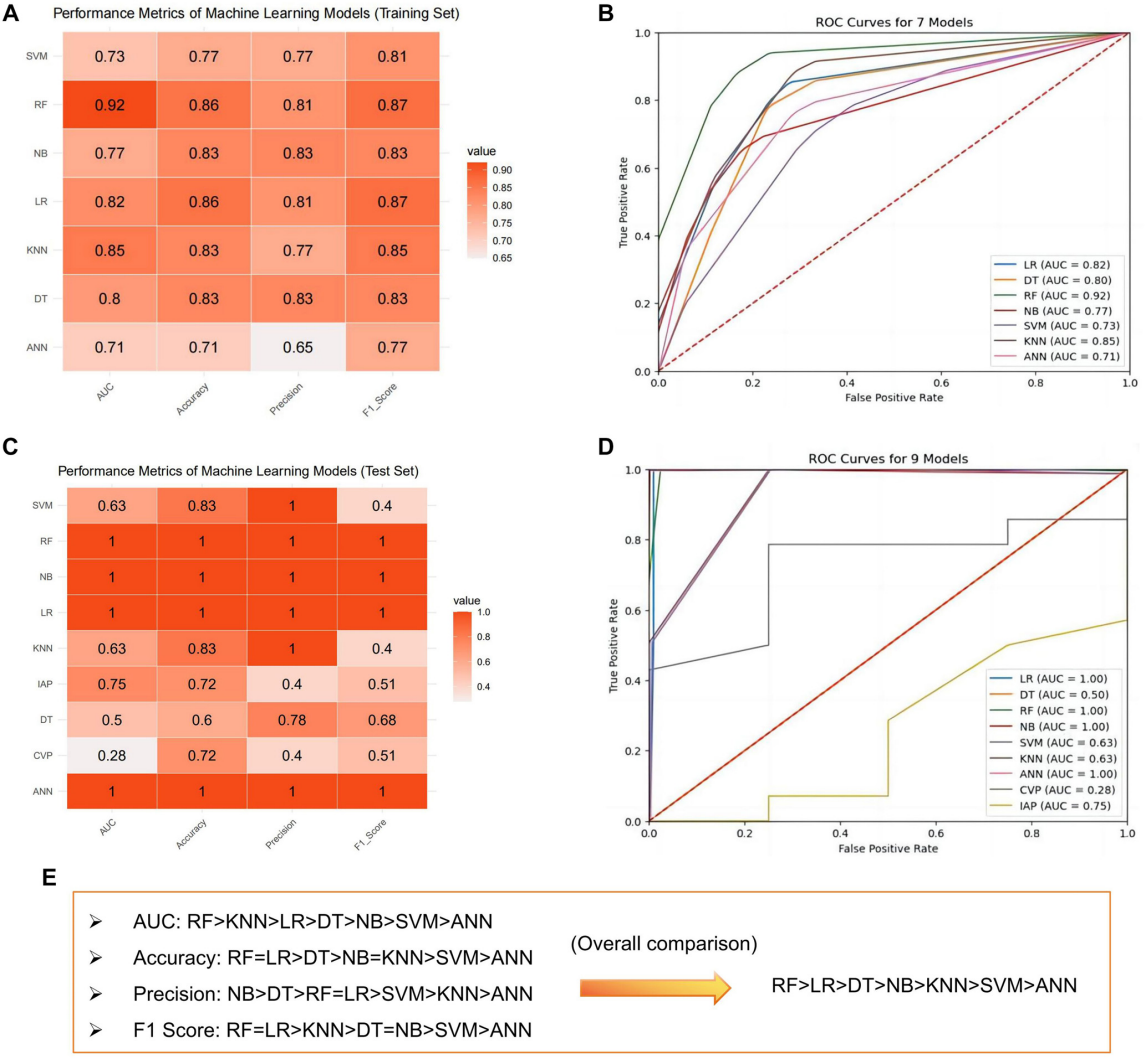
**Training and test results of the HI prediction model.** (A) Performance metrics of the training set for the HI prediction model constructed using seven machine learning algorithms; (B) ROC curve of the HI prediction model on the training set; (C) Performance metrics of the test set for the HI prediction model constructed using seven MLalgorithms; (D) ROC curve of the HI prediction model on the test set; (E) Flowchart comparing and selecting algorithms, providing a comprehensive overview of the performance of ML models based on training and test sets. The reasons for selecting the RF and LR models are highlighted, as these two models demonstrated superior performance on key metrics. LR: Logistic regression; DT: Decision tree; RF: Random forest; NB: Naive Bayes; SVM: Support vector machine; KNN: K-nearest neighbors; ANN: Artificial neural network; CVP: Central venous pressure; IAP: Intra-abdominal pressure; HI: Hemodynamic instability; ROC: Receiver operating characteristic; ML: Machine learning.

In this study, we applied seven ML algorithms—LR, DT, NB, SVM, KNN, RF, and ANN—to predict HI. We evaluated their performance using metrics, such as AUC, accuracy, precision, and F1 scores for both training and validation datasets. Figure 4 summarizes the performance of all algorithms. Figure 4A and 4B presents the AUC, accuracy, precision, and F1 scores for each model. Among these, the RF model performed best in the training set, achieving an AUC of 0.92, accuracy of 0.86, precision of 0.81, and an F1 score of 0.87. The LR model also performed well, with an AUC of 0.82, accuracy of 0.86, precision of 0.81, and an F1 score of 0.83. Both the C5.0 DT and NB models showed comparable performance, each achieving an AUC of 0.80 and 0.77, an accuracy and precision of 0.83, and an F1 score of 0.83. The KNN model achieved an AUC of 0.85, accuracy of 0.83, precision of 0.77, and an F1 score of 0.85. In contrast, the SVM model demonstrated an AUC of 0.73, accuracy of 0.77, precision of 0.77, and an F1 score of 0.81. The ANN model had the lowest performance, with an AUC of 0.71, accuracy of 0.71, precision of 0.65, and an F1 score of 0.77. Overall, the RF model consistently outperformed the others across all metrics, indicating its superior predictive capability for HI. The calibration plot (Figure S1A) demonstrated good agreement between the RF model’s predicted probabilities and actual outcomes, particularly in medium- to high-risk ranges. DCA (Figure S1B) further highlighted the RF model’s net benefit across varying thresholds, showing a significantly greater net benefit compared to the “treat all” and “treat none” strategies in moderate threshold ranges. These results underscore the RF model’s clinical utility and its potential application in practice.

**Figure 5.**
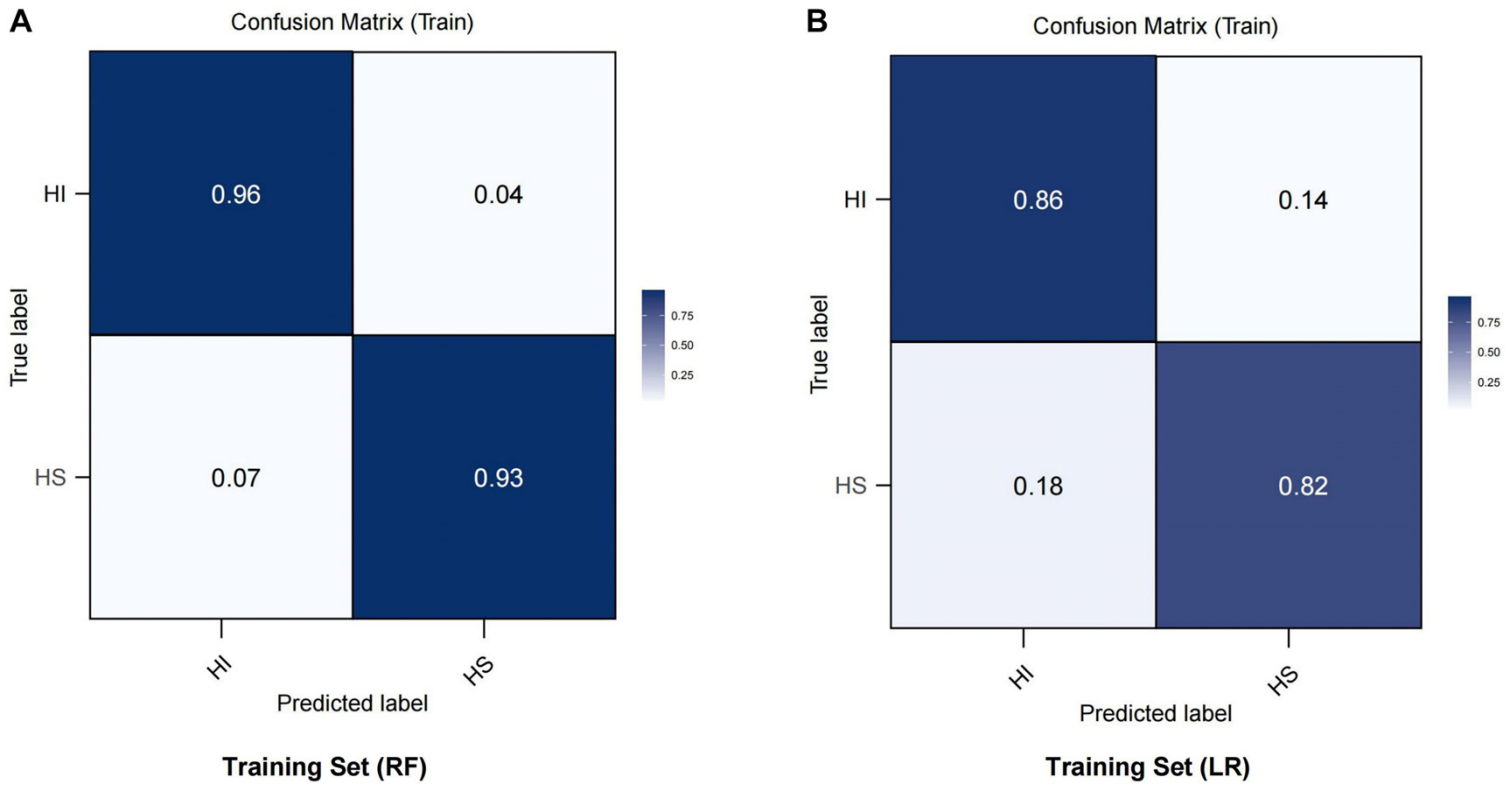
**Confusion matrix results of two different machine learning models on the training set.** (A) Confusion matrix of the RF model. The horizontal axis represents the predicted labels, and the vertical axis represents the actual labels. HI denotes hemodynamically unstable patients, and HS denotes hemodynamically stable patients. The model’s prediction accuracy for HI and HS is 0.96 and 0.93, respectively. (B) Confusion matrix of the LR model, with the same horizontal and vertical axes as above. The model’s prediction accuracy for HI and HS is 0.86 and 0.82, respectively. LR: Logistic regression; RF: Random forest; HI: Hemodynamic instability.

**Figure 6.**
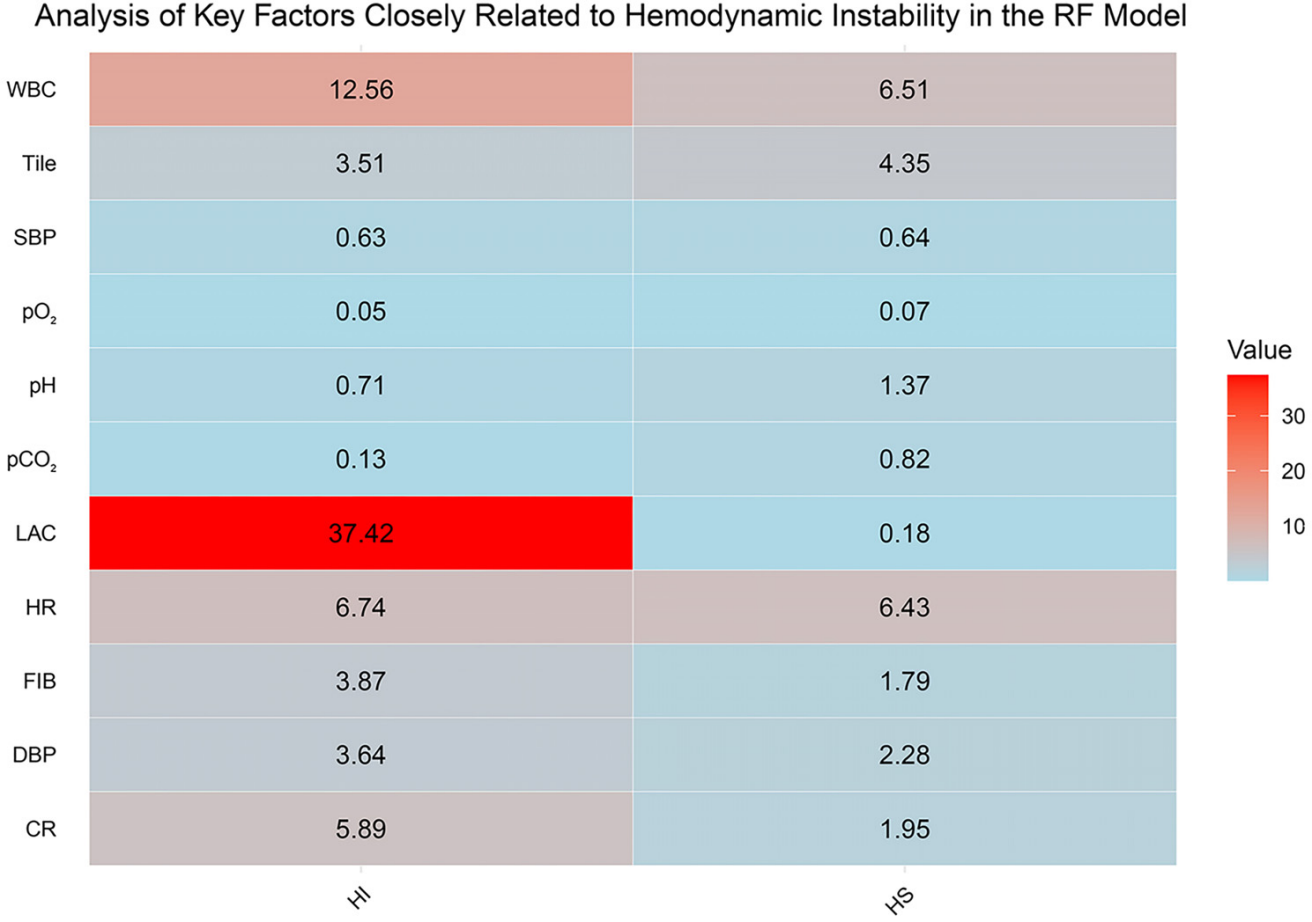
**Key factors closely associated with HIidentified by the RF model.** This heatmap displays key factors identified by the RF model closely associated with hemodynamic instability (HI vs HS). The values represent the average levels of different clinical indicators under two conditions (hemodynamically unstable vs stable), with color intensity reflecting the magnitude of these values. Red areas highlight significantly elevated lactate levels (LAC) and WBC in hemodynamically unstable patients, indicating their importance as risk factors. The clinical indicators include HR, SBP, DBP, WBC, Tile Classification (Tile), FIB, CR, pH, pCO_2_, pO_2_, and LAC. RF: Random forest; HI: Hemodynamic instability; WBC: White blood cell count; HR: Heart rate; SBP: Systolic blood pressure; DBP: Diastolic blood pressure; FIB: Fibrinogen; CR: Creatinine; pH: Hydrogen ion concentration; pCO_2_: Partial pressure of carbon dioxide; pO_2_: Partial pressure of oxygen; LAC: Lactic acid.

The test set results mirrored those of the training set (Figure 4C and 4D). The RF model performed best, achieving an AUC of 1.00, demonstrating exceptional diagnostic capability, followed by the LR algorithm. Based on a comprehensive comparison of the seven algorithms (Figure 4E), we focused our discussion on the RF and LR models. To further evaluate the predictive performance of these models, we analyzed confusion matrices for the RF and LR models on the training set (Figure 5A and 5B). The RF model achieved a classification accuracy of 96% for hemodynamically unstable patients and 93% for stable patients. These findings indicate the RF model’s strong capability in identifying high-risk patients, making it highly valuable for clinical prediction and real-time monitoring systems. The LR model, while less accurate, achieved a classification accuracy of 86% for unstable patients and 82% for stable patients, suggesting its potential utility in resource-limited settings. In summary, the RF model demonstrated superior performance in predicting HI, but the LR model remains a viable clinical support tool. These results suggest that ML models, especially the RF model, can improve the efficiency and effectiveness of managing critically ill patients by enabling earlier identification and intervention for high-risk individuals. This could potentially lead to better outcomes for patients, particularly those with conditions such as closed PFs. Through factor analysis of the RF model (Figure 6), we identified several key indicators strongly associated with HI, including WBC and LAC levels. Elevated lactate levels were particularly notable, with levels above 3.7 (OR ═ 1.178, 95% CI: 1.114–1.259, *P* ═ 0.043) identified as an independent risk factor. These findings provide valuable clinical insights, supporting early identification and intervention in patients at risk of HI.

### Internal and external validation of mortality prediction models

**Figure 7.**
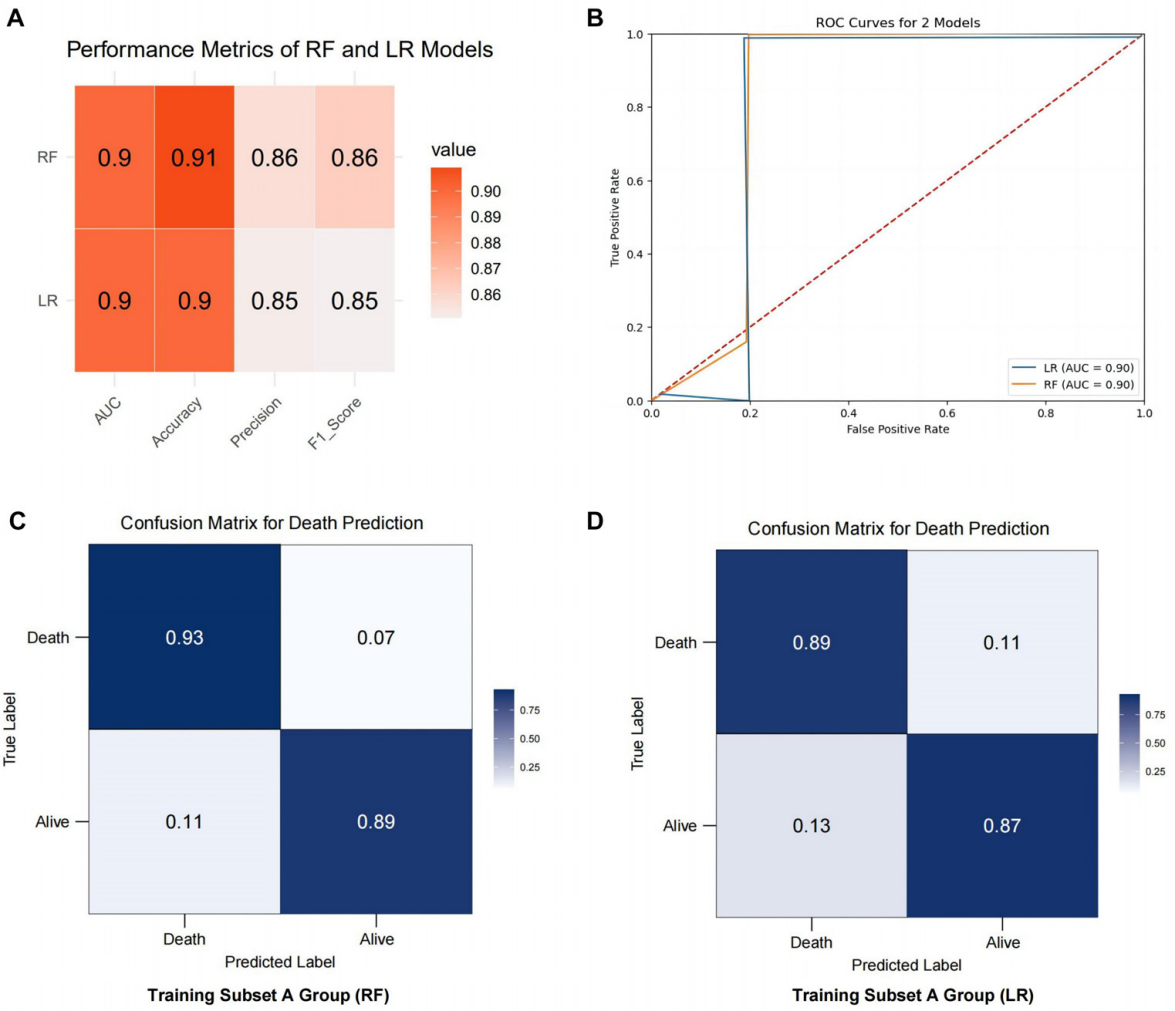
**Performance analysis of mortality prediction models built with RF and LR algorithms.** (A) Comparison of performance metrics, including AUC, accuracy, precision, and F1 score, for mortality risk prediction models for HI patients developed using training subset Group A; (B) ROC curves for RF and LR models, illustrating the relationship between true positive rate and false positive rate at various thresholds; (C) Confusion matrix for mortality prediction in the RF model on training subset Group A; (D) Confusion matrix for mortality prediction in the LR model on training subset Group A. RF: Random forest; LR: Logistic regression; HI: Hemodynamic instability; AUC: Area under the ROC curve; ROC: Receiver operating characteristic.

This study focuses on validating mortality prediction models based on RF and LR algorithms. Figure 7 presents a comparison of the two algorithms in predicting mortality risk for HI patients, evaluated using performance metrics, such as AUC, accuracy, precision, F1 score, and a confusion matrix. The results show that the RF model performed best on the training set, achieving an AUC of 0.90, accuracy of 0.91, precision of 0.86, and an F1 score of 0.86. Similarly, the LR model demonstrated strong performance with an AUC of 0.90, accuracy of 0.90, precision of 0.85, and an F1 score of 0.85 (Figure 7A). The ROC curve (Figure 7B) confirms that both models have an AUC of 0.90, indicating robust discriminative power in predicting mortality risk.

**Figure 8.**
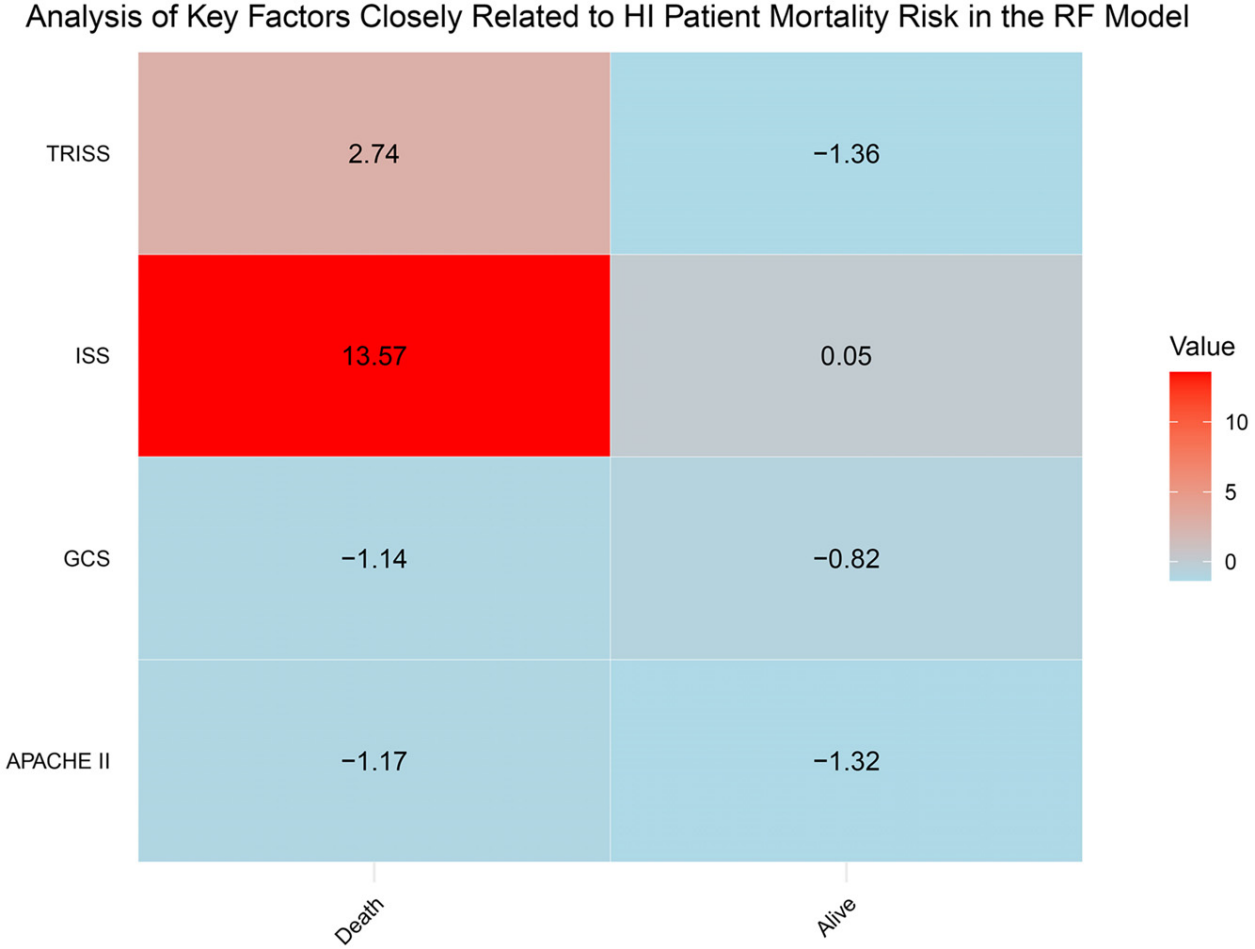
**Key factors closely associated with mortality risk in HI patients analyzed using the RF model.** This figure displays the importance of four main factors related to mortality risk in HI patients within the RF model: TRISS, ISS, GCS, and APACHE II. Each cell represents the weight of a specific factor in predicting mortality (red bars) or survival (blue bars). The factors include the ISS, GCS, TRISS, and APACHE II. HI: Hemodynamic instability; GCS: Glasgow Coma Scale; ISS: Injury Severity Score; TRISS: Trauma and Injury Severity Score; APACHE II: Acute Physiology and Chronic Health Evaluation; RF: Random forest.

The confusion matrix analysis reveals that, within the training subset Group A, the RF model achieved a classification accuracy of 93% for deceased HI patients and 89% for surviving HI patients (Figure 7C). This underscores the RF model’s high effectiveness in identifying potential mortality risk in HI patients, highlighting its utility for clinical prediction and real-time monitoring systems. In comparison, the LR model achieved a classification accuracy of 89% for deceased HI patients and 87% for surviving HI patients (Figure 7D). Overall, the RF model outperformed the LR model for this specific task, demonstrating higher classification accuracy. Further analysis identified a significant risk factor (Figure 8): an ISS score greater than 13 (OR ═ 1.088, 95% CI: 1.024–1.259, *P* ═ 0.043). This finding suggests that an elevated ISS score is associated with increased mortality risk. In conclusion, this study demonstrates that the RF model outperformed the LR model in predicting mortality risk for patients with PFs, and factor analysis highlights the importance of ISS scores in assessing patient outcomes.

**Figure 9.**
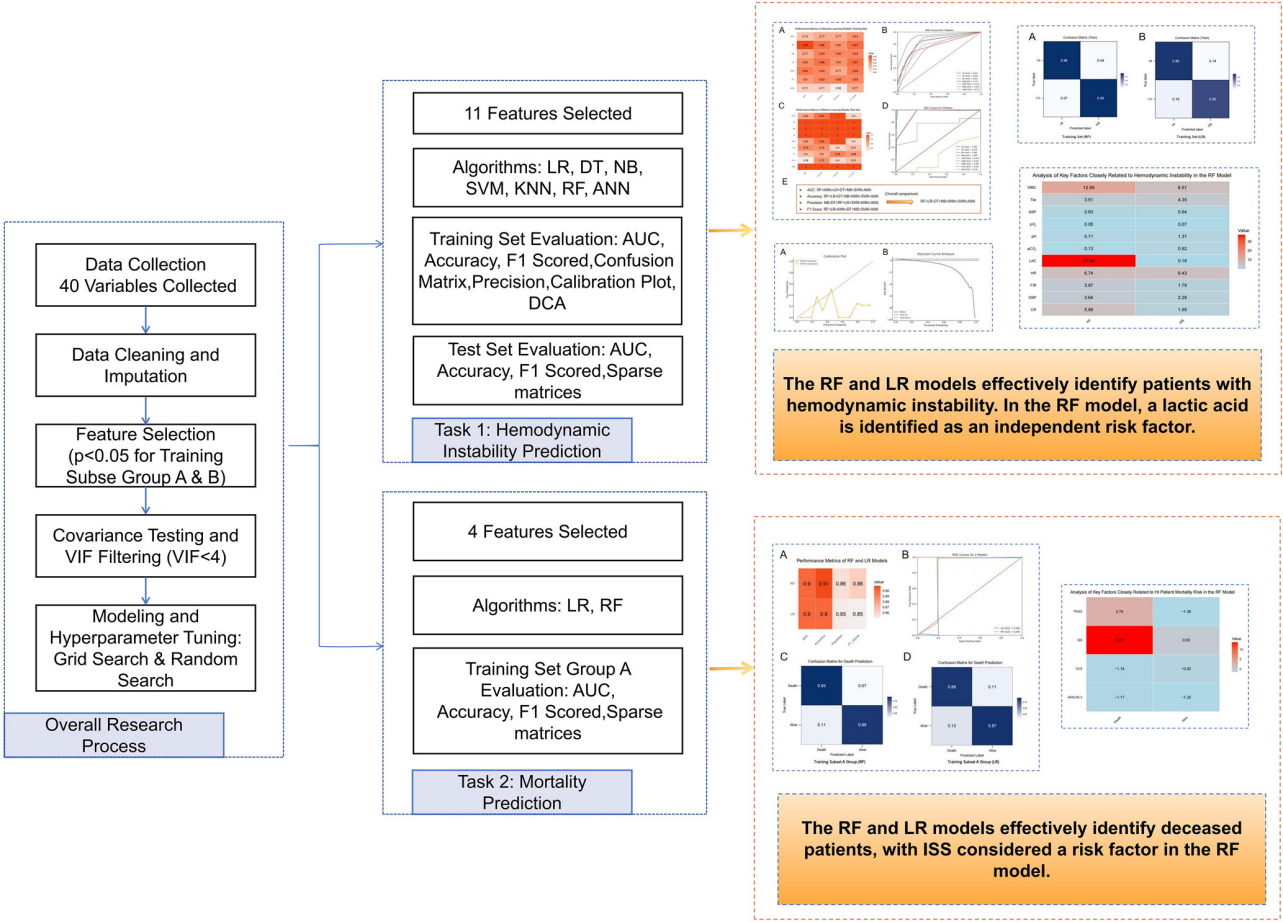
**Research mechanisms for predictive models of hemodynamic instability and mortality risk.** SVM: Support vectormachine; KNN: K-nearest neighbors; RF: Random forest; ANN: Arti cial neural network; VIF: Variance in ation factor; AUC: Area under the ROC curve; DT: Decision tree; LR: Logistic regression.

## Discussion

This study developed and validated a novel predictive model using seven ML algorithms to forecast closed PFs combined with HI. The model demonstrated superior predictive capability compared to traditional measures, such as CVP and IAP, underscoring the significant potential of ML methods in medical research. Unlike prior studies, which primarily focused on patient characteristics, predictive algorithms, or model performance [37–40], this innovation offers a more comprehensive approach. In this study, seven ML algorithms—LR, DT, NB, SVM, KNN, RF, and ANN—were used to construct the predictive model, which is a less common approach in comparative research. A dataset comprising 40 clinical indicators, surpassing the scope of previous studies, was analyzed. To ensure robustness, a five-fold cross-validation method was employed [41], which utilized all training data for model training and testing, effectively minimizing overfitting risks [42–44]. Regarding model performance, RF and LR exhibited strong predictive capabilities in both the training and test datasets [45–47]. RF, in particular, excelled due to its ability to handle high-dimensional data and nonlinear relationships, achieving favorable results even without parameter tuning [48–50]. The RF algorithm proved effective in identifying patients with closed PFs combined with HI by analyzing variables, such as HR, SBP, DBP, WBC, Tile classification, FIB, CR, pH, pCO_2_, pO_2_, and lactate (LAC). Notably, a lactate level above 3.7 (OR ═ 1.178, 95% CI: 1.114–1.259, *P* ═ 0.043) was identified as an independent risk factor, while an ISS greater than 13 was also highlighted as a potential risk factor (OR ═ 1.088, 95% CI: 1.024–1.259, *P* ═ 0.043). These findings further validate the use of lactate levels in predicting HI.

Currently, the clinical assessment of HI in patients with closed PFs relies primarily on blood pressure and biochemical indicators [51], while mortality risk is typically evaluated using ISS and lactate levels at admission [52 ]. However, assessing HI and mortality risk in these cases is a complex process that involves multiple factors. In this study, RF and LR models were applied to predict mortality risk in HI patients, with RF demonstrating superior performance across key metrics. The analysis identified an ISS greater than 13 as a significant risk factor for mortality, while Tile C, the most severe classification of PFs, was used to assess fracture severity. Other important factors, including HR, LAC, and FIB, were also found to be associated with HI.

The models and indicators from this study could assist clinicians in assessing the prognosis of closed PF patients and potentially improving survival rates through targeted interventions. Furthermore, these models could be integrated into web pages and applications, facilitating clinical use and enabling data collection to refine and optimize predictive accuracy. The significant clinical value of this study lies in its innovative ML-based approach, which proposes a predictive model for assessing HI and mortality risk in patients with closed PFs. This model aims to enhance timely diagnosis, treatment, and overall clinical management, improving outcomes for this critical condition that poses a serious threat to survival. By leveraging various ML algorithms, the study more accurately identifies high-risk patients, providing essential data support for prompt and effective clinical decision-making, especially in emergencies. This data-driven approach is particularly valuable in resource-limited settings, such as pre-hospital emergency care, and in cases where traditional invasive diagnostic methods may not be suitable, such as for pregnant women and children. Additionally, it aids in optimizing the allocation of medical resources, thereby improving treatment efficiency and overall patient outcomes.

Nevertheless, the study has some limitations. Due to its retrospective nature, there is a potential for selection bias and limited data traceability. To address the challenges posed by the small sample size, we implemented several measures to reduce the risk of overfitting, including cross-validation and regularization techniques. It is important to note that some models achieved a perfect AUC value (1.00) on the test set. While this demonstrates model efficiency, it may also suggest overfitting during training. Future studies should adopt stricter validation approaches, such as using independent test sets or enhanced cross-validation methods, to minimize this risk. Additionally, the confusion matrix results were derived from a small subset (training subset A), which restricts the generalizability of the findings. Future research should validate these models on a larger, more diverse patient population to ensure that the findings carry meaningful clinical value. The small sample size may also result in high variance, leading to inconsistent model performance across different sample groups. Furthermore, since the data were sourced from a single medical center, this may limit the model’s broader applicability and generalization potential.

The small sample size and single-center data source may limit the model’s generalizability and scalability. A small dataset could lead to imbalances in variables, such as gender and age, reducing the model’s real-world applicability. These imbalances may increase prediction bias for minority classes, compromise the model’s interpretability in predicting HI and mortality risk in patients with closed PFs, and heighten uncertainty in its predictions. As a result, the model’s utility in guiding clinical diagnosis and treatment may be reduced. Furthermore, the training and validation processes did not account for patients’ resuscitation responses, potentially affecting prediction accuracy. Future studies should address these limitations by including larger sample sizes and multicenter data to enhance the model’s generalizability and performance.

We observed high collinearity among certain clinical indicators, which could affect the model’s robustness and variable selection. To address this, we applied regularization methods, such as Lasso regression, to reduce model complexity and enhance prediction accuracy. These techniques help minimize overfitting by penalizing less important coefficients and selecting the most predictive variables. In future studies, we plan to test model robustness using different data-splitting strategies and additional independent datasets. Additionally, we will explore ensemble learning methods to mitigate overfitting associated with individual models. To address class imbalance, we applied SMOTE and undersampling strategies in the training set, which effectively improved the model’s performance during training. This approach is appropriate, as avoiding data balancing techniques in the test set preserves authenticity in evaluation and prevents artificially distorting the model’s generalization to new data. Future research will examine the impact of these techniques on model generalization and explore more balanced strategies for handling data imbalance. This will ensure the model achieves high accuracy and reliability across diverse clinical settings.

In addition, future research will aim to extend the application of these ML models to specialized contexts and populations, such as pre-hospital emergency care and pregnant women. Experimental testing, including animal studies or clinical trials, will be conducted to assess the effects and underlying mechanisms of these influencing factors. This will allow for predictive testing and provide deeper insights into their impact. Ultimately, these efforts seek to translate predictive models into practical tools for clinical use, such as interactive web pages and applications, thereby enhancing both the quality and efficiency of medical practice.

## Conclusion

This study employed seven ML algorithms, including RRF and LR, to predict HI and mortality risk in patients with closed PFs (Figure 9). The results demonstrated that the RF algorithm outperformed the others, excelling in metrics, such as the confusion matrix, mean AUC, accuracy, precision, and F1 score. An in-depth analysis of patient data revealed that lactate levels were a key factor influencing HI, while an ISS greater than 13 emerged as a significant predictor of mortality risk. Although the study faced limitations, such as a small sample size and potential data accuracy issues, its findings provide valuable decision-support insights for clinicians and pave the way for future research and clinical applications.

## Supplemental data

**Figure S1.**
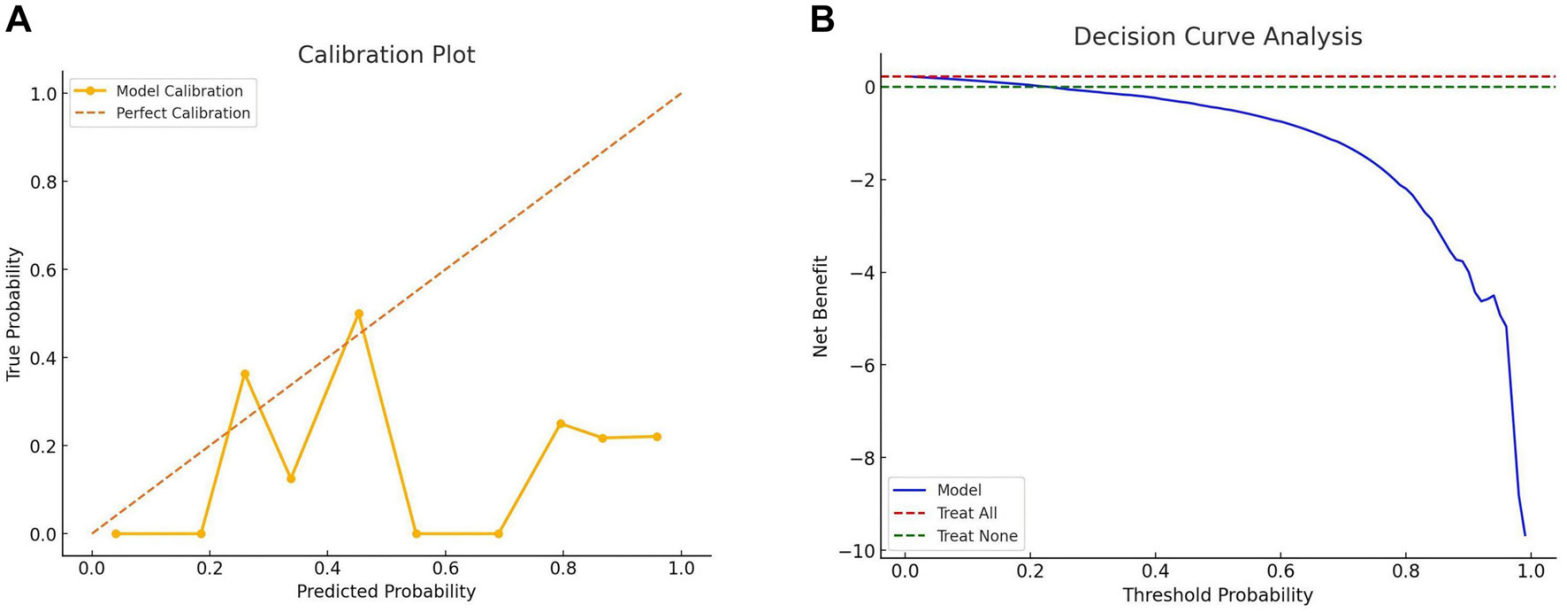
**Calibration and DCA of the RF model on the training set.** (A) The calibration plot shows the consistency between the predicted probabilities of the RF model and the actual observed probabilities. The diagonal line represents perfect calibration; the closer the models curve is to this line, the more consistent its predictions are with actual outcomes. In this study, the model demonstrates good calibration across most probability ranges, especially in the medium- to high-risk ranges. (B) The decision curve illustrates the net benefit of the RF model at different thresholds. DCA evaluates the model’s net benefit across various threshold values. The blue curve represents the model’s net benefit, the red dashed line represents the “treat all” strategy, and the green dashed line represents the “treat none” strategy. RF: Random forest; DCA: Decision curve analysis.

## Data Availability

All data can be provided as needed.
